# Continuous beta-2 microglobulin–based clearance highlights superiority of high-Dose HDF over high-flux HD in predicting outcomes

**DOI:** 10.1038/s41598-025-07497-2

**Published:** 2025-07-01

**Authors:** Bernard Canaud, Andrew Davenport, Marion Morena-Carrere, Mailis Amico, Nicolas Molinari, Jean-Paul Cristol

**Affiliations:** 1 Nephrology-Dialysis, Intensive Care Unit -University of Montpellier, 9 Rue Des Carmelites, 34090 Montpellier, France; 2 Department of Renal Medicine, UCL, Royal Free Hospital, University College London, London, UK; 3 Department of Biochemistry and Hormonology, PhyMedExp, University of Montpellier, INSERM, CNRS, University Hospital Center of Montpellier, Montpellier, France; 4 Clinical Research and Epidemiology Unit, University Hospital Center of Montpellier, University of Montpellier, Montpellier, France

**Keywords:** End Stage Kidney Disease, Kidney Replacement Therapy, Beta-2-Microglobulin, Patient outcome, Mortality, Diagnostic markers, Prognostic markers, Nephrology

## Abstract

Recent studies suggest that high-dose hemodiafiltration (HDF) may reduce mortality more effectively than high-flux hemodialysis (HD), though the mechanisms remain unclear. Traditional metrics such as Kt/V and convective volume do not fully capture overall dialysis efficiency. This study proposes a novel approach using circulating beta-2-microglobulin (ß2M) levels to estimate an equivalent Continuous Dialytic Clearance (eCDC_ß2M_), reflecting an equivalent glomerular filtration rate. Using data from the FRENCHIE study, we calculated eCDC_ß2M_ and assessed its association with patient outcomes, including all-cause and cardiovascular mortality, in comparison with traditional dialysis dose metrics. Our analysis showed that HDF achieved higher treatment efficiency than high-flux HD, with a mean increase of + 1.5 ml/min in eCDC_ß2M_. Moreover, eCDC_ß2M_ demonstrated superior predictive value for mortality risk compared to Kt/V. These findings support eCDC_ß2M_ as a meaningful and physiologically relevant measure of dialysis efficiency and adequacy. By better reflecting the continuous function of the native kidney, this approach may improve patient stratification and outcome prediction across all forms of kidney replacement treatment schedule. Further validation in independent patient cohorts is warranted.

## Introduction

Several recent studies, including the CONVINCE trial and the updated individual patient data meta-analysis, have demonstrated that high-dose hemodiafiltration (HDF) is superior to high-flux hemodialysis (HD) in reducing all-cause mortality risk for end-stage kidney disease (ESKD) patients treated by dialysis^1–5^. However, the exact mechanisms and biomarkers supporting these benefits remain debated or hypothetical^6^. While the removal of larger uremic toxins is a potential explanation, quantifying the true efficiency of these kidney replacement therapies is challenging in clinical practice^7–10^.

Current approaches to quantifying the efficiency of kidney replacement treatment rely on surrogate markers. In hemodialysis, a diffusive-based modality, Kt/V, a dimensionless ratio, estimates the sessional fractional urea clearance^11–13^. In HDF, a convective-based modality, the convective volume delivered per session serves as a proxy of middle and large sized uremic solute clearances^14–20^. These methods have limitations, as highlighted by the CONVINCE study, which did not report any specific biomarker to support the beneficial effects of HDF except for these two surrogate markers of small and middle-sized solute clearance^3^. Combining clearances of small and middle to large molecular weight uremic markers will provide a more comprehensive approach to quantify overall efficiency across a larger spectrum of uremic compounds and all dialysis modalities^21,22^. Therefore, a more objective measure reflecting overall kidney replacement therapy efficiency and more closely resembling the continuous function of native kidney function is needed.

In this context, beta-2 Microglobulin (ß2M) is potentially a highly valuable biomarker for this purpose^23,24^. Established in renal physiology, circulating ß2M levels or ß2M clearances can be used to estimate glomerular filtration rate (GFR)^23,25^. This characteristic makes it a promising biomarker to quantify overall kidney replacement therapy efficiency, including residual kidney function^23–26^. Furthermore, circulating levels of ß2M go beyond kidney function or dialysis efficiency, as ß2M may also serve as a cardiovascular risk marker^27^, relevant not only for chronic kidney disease patients but also for those with other acute or chronic disease conditions^28–31^.

In this study, our aim was to explore an innovative approach to estimating the equivalent of a continuous dialytic clearance based on circulating levels of ß2M (eCDC_ß2M_). To achieve this, we utilized the dataset from the French HDF study (FRENCHIE trial), which collected plasma/serum samples at various time points during the study and conducted centralized laboratory testing on various biomarkers, including ß2M, as part of the two-year follow-up^32^. Subsequently, we calculated an equivalent of glomerular filtration rate namely equivalent Continuous Dialytic Clearance (eCDC_ß2M_) based on ß2M concentrations established by the CKD-EPI consortium^23,33,34^. Additionally, we assessed the effect of this eCDC_β2M_ equivalent of glomerular filtration rate on patient survival and compared this to conventional urea derived clearances by evaluating the predictive effect on patient survival in ESKD patients treated by both hemodialysis and hemodiafiltration.

## Material and methods

### Study design: the FRENCHIE trial

This study is a post hoc analysis of the FRENCHIE trial, which was a multicenter prospective randomized trial with open labeling^32^. The original study enrolled 381 elderly (over 65 years old) prevalent chronic hemodialysis (HD) patients, out of which 286 were studied as they had blood samples stored for post hoc analysis. These patients were divided equally (1:1 ratio) into two groups: one receiving high-flux HD and the other receiving postdilution HDF. The primary endpoint was to assess patient outcome (intradialytic morbidity) according to the dialysis modality from day 30 to day 120. Secondary outcomes included biological markers associated with cardiovascular disease, hospital admissions for any cause and those specifically due to cardiovascular conditions, death from any cause and cardiovascular-related deaths over a two-year follow-up period.

Detailed steps for calculating eCDC from time averaged β2M (ß2M_TAC_) concentrations and kidney replacement therapy efficiency indicators are provided in the appendix. Postdialysis β2M was adjusted to align with a two-compartment model by accounting for extracellular volume contraction and incorporating a 60-min postdialysis equilibration phase, as described by Ward et al.^35^. The midweek time-averaged concentration of β2M was calculated using the pre and post hemodialysis concentrations from the mid-week session.

### Calculation of kidney replacement therapy efficiency indicators

Percent reduction (PR) of urea was calculated individually as:

1 $${PR}_{Urea}= \left(1-{Urea}_{post}/{Urea}_{pre}\right) x 100$$

where urea_pre_ and urea_post_ refer to pre and post dialysis urea concentrations in mmol/L.

Single pool Kt/V urea was calculated individually using the Daugirdas formula:

2 $${spKtV}_{urea}=- Ln\left({Urea}_{post}/{Urea}_{pre}-0.008.{t}_{HD}\right)+ \left(4-3.5.\left({Urea}_{post}/{Urea}_{prs}\right).\left({BW}_{pre}- {BW}_{post}/{BW}_{post}\right)\right)$$

where Urea_pre_ and Urea_post_ refer to pre and post dialysis urea concentrations in mmol/L, BW_pre_ and BW_post_ for pre and post body weight in kg and t_HD_ for treatment duration in hours. Postdialysis blood samples were taken after reducing blood pump for about 3 min.

### Calculation of equivalent Continuous Dialytic Clearance from ß2M (${eCDC}_{\ss 2M}$) circulating concentrations

To estimate the equivalent continuous dialytic clearance (${eCDC}_{\ss 2M}$), we used two midweek ß2M plasma concentration measurements (pre and post dialysis) collected during the FRENCHIE trial, applying an emulation of a two-compartment kinetic model of ß2M. To correct for extracellular volume contraction due to ultrafiltration, the post-dialysis ß2M concentrations were adjusted using Bergström’s formula^36^. Additionally, to account for the compartmentalization effect and 60-min rebound, the Tattersall equation was applied^37,38^. Pre- and post-dialysis ß2M concentrations (ß2M_pre_ and ß2M_post_) were measured using the COBAS Roche platform, with the Tina-quant β2-Microglobulin assay performed on an automated analyzer (Cobas 8000, Roche Diagnostics, Meylan, France).

Corrected postdialysis ß2M concentrations for hemoconcentration:

The correction accounts for extracellular volume changes due to net ultrafiltration and is calculated as:

3 $${\ss 2M}_{post-cor}={\ss 2M}_{post}/\left(1+WL/0.2 {x BW}_{post}\right)$$

where, *WL* represents the weight loss during dialysis (in kg) used as proxy for ultrafiltration volume; $O.2 . {BW}_{post}$ estimates the extracellular volume (ECV) in liters, based on post-dialysis body weight; the ratio $\left(WL/0.2 x {BW}_{post}\right)$ serves as a proxy for hemoconcentration (ECV contraction) resulting from fluid removal.

Postdialysis ß2M concentration corrected for the compartmentalization effect (two pool model):

4 $${\ss 2M}_{post-eq}= {\ss 2M}_{pre} {\left({\ss 2M}_{post-cor}/{\ss 2M}_{pre}\right)}^{{t}_{HD}/\left({t}_{HD}+60\right)}$$

This used Tattersall’s equation, which estimates the equilibrated postdialysis ß2M concentration by accounting for a 60-min rebound period, thereby reflecting the compartmentalization effect as reported in Ward’s two-pool ß2M kinetic model^39^.

The percent reduction of ß2M (${PR}_{\ss 2M}$), in%, was calculated individually as:

5 $${PR}_{\ss 2M}= \left(1- {{\ss 2M}_{post-eq}/\ss 2M}_{pre}\right) x 100$$

The Time Averaged Concentration of ß2M ${(\ss 2M}_{TAC}$ ), in mg/l was calculated as follows:

6 $${\ss 2M}_{TAC}= \left({\ss 2M}_{pre}- {\ss 2M}_{post.eq}\right)/Ln\left({\ss 2M}_{pre}/{\ss 2M}_{post.eq}\right)$$

This formula uses the mid-week logarithmic mean value of ß2M as the best estimate of its concentration over the dialysis cycle. This approach has been recently validated in a direct dialysate quantification study^40^:

Effective ß2M clearance (${K}_{\ss 2M}$) in ml/min, was calculated as:

7 $${K}_{\ss 2M}=[ECV x Ln \left({\ss 2M}_{pre}/{\ss 2M}_{post.eq}\right)]/{t}_{HD} x 1000$$

where ECV refers to extracellular volume as ${O.2 x BW}_{post}$ in liters, ${\ss 2M}_{pre}$ and ${\ss 2M}_{post.eq}$ are the predialysis and postdialysis ß2M concentrations respectively, in mg/l and ${t}_{HD}$ is the dialysis treatment duration in minutes.

ß2M mass removal (${\ss 2M}_{MR}$), expressed in mg per session was calculated as:

8 $${\text{\ss }2M}_{MR}=\left({\text{\ss }2M}_{TAC} x {K}_{\text{\ss }2M} x {t}_{HD}\right) /1000$$

with ${\ss 2M}_{TAC}$ in mg/l, ${K}_{\ss 2M}$ in ml/min and ${t}_{HD}$ in minutes.

Calculation of estimated Continuous Dialysis Clearance of ß2M (${eCDC}_{\ss 2M}$), expressed in ml/min, from the time-averaged concentration ( ${\ss 2M}_{TAC}$)

9 $${eCDC}_{\ss 2M}=133 x {{\ss 2M}_{TAC}}^{- 0.854}$$

${\ss 2M}_{TAC}$ and ${eCDC}_{\ss 2M}$ were calculated individually for the entire population (including both HDF and HD patients) and averaged over the two year-follow-up period. Please note that two to three values per patient were used for these calculations.

### Endpoints

Patient all-cause and cardiovascular mortality were defined as the time from study inclusion to death from all-cause and the time from study inclusion to death from cardiovascular cause, respectively. Patient deaths were classified as either having occurred or not from all causes at the completion of the study.

### Ethics

After approval by the ethics committee at Montpellier University Hospital, the FRENCHIE trial was registered with the French Ministry of Health and Solidarity ("Ministère de la Santé et des Solidarités"). Funding was exclusively provided by the Ministry of Health through a program called " Hospital Clinical Research Project" (PHRC) and registered as Clinical Trial ID# NCT01327391. All procedures were conducted in strict accordance with relevant guidelines and regulations. Written informed consent was obtained from all participating patients to ensure compliance with ethical standards.

### Statistical analysis

Descriptive statistics of patient characteristics, dialysis prescription and dialysis efficiency parameters were obtained for all groups (HDF or high-flux HD) and analyzed using R software. Quantitative variables were described using medians and interquartile ranges (25th and 75th percentiles) and compared between groups using the Wilcoxon rank-sum test. Qualitative variables were described using frequency counts and percentages. Comparison between groups was performed using either Fisher exact test or the Chi-square test for qualitative variables, depending on the expected counts. Dialysis parameters were collected at baseline, 12 and 24 months. The average over these time points was computed for each parameter and used for descriptive analysis. eCDC_ß2M_ was compared between treatment modalities (HDF vs. HD) using the Wilcoxon rank-sum test.

The survival curves for all-cause mortality comparing eCDC_β2M_ values categorized into three groups based on tertiles from the total cohort were analyzed using Kaplan–Meier plots. The Peto and Peto log-rank test for crossing curves was used to compare survival between groups. Cardiovascular mortality for eCDC_β2M_ groups was estimated based on a competing risk analysis considering other types of death as competing events. Cumulative incidence functions for cardiovascular mortality were calculated and the Gray test used to compare curves.

The effect of eCDC_ß2M_ and Kt/V on patient death for the total cohort was analyzed using a multivariate Cox proportional hazards model. In order to explore the potential effect of other factors affecting the risk of death; age, sex and comorbidities (diabetes, hypertension, heart failure, ischemic cardiac disease and arrhythmia) were included in the model, alongside eCDC_ß2M_ or Kt/V. A multivariate model selection based on a step backward approach was performed and the final model only included statistically significant variables.

## Results

A total of 286 patients from the original FRENCHIE cohort (n = 386) were included in the present analysis, 144 were treated by HD and 142 by HDF. The 286 patients were selected on the availability of a consistent number of blood samples, all of which were collected and processed centrally. Standardized ß2M measurements were performed in a uniform and centralized manner. The main findings of the study are presented and organized into four sections:

This section presents the baseline characteristics of the study population, followed by an analysis of treatment prescription and efficiency of the two dialysis modalities.

Table 1 details the baseline characteristics of the elderly stage 5 CKD dialysis (CKD5D) population (median age 76.2 years, 60.5% male, median post-dialysis body weight 68.7 kg) evenly divided between high-flux HD and hemodiafiltration (HDF). The two treatment groups were well balanced and did not show statistically significant difference in terms of comorbidities; 39.0% had diabetes mellitus, 76.6% hypertension, 14.9% congestive heart failure, 35.8% ischemic cardiac disease, 12.1% arrhythmia, 2.8% heart valve disease, and 2.5%-6.8% had peripheral vascular disease, along with similar sex distribution and anthropometric characteristics including body weight. Pre- to post-dialysis body weights were averaged over multiple time points to assess session weight loss as a surrogate of net ultrafiltration volume. Inflammatory status assessed using high-sensitivity CRP showed no significant differences (p 0.311) between groups (median [Q1; Q3] CRP level: 5.1 [2.6;12.6] mg/L in HD vs. 7.0 [3.0;15.7] mg/L in HDF). Importantly, all patients were prevalent dialysis patients, with a mean dialysis vintage of 4.6 years in the HD group and 5.0 years in the HDF group. Residual kidney function was considered negligible across the entire cohort, as patients had no significant residual urine output at baseline.

**Table 1:** Baseline characteristics of elderly stage 5 CKD patients on high-flux HD or HDF, showing well-balanced groups in terms of demographics, comorbidities, body weight, dialysis vintage, and inflammatory status.

Characteristics	Total	HD	HDF	Test	p-value
Patients (n)	286	144	142		
Age (years)	76.2	76.2	76.2	Wilcoxon	0.821
Median [Q1_Q3]	[71.1;80.9]	[71.1;81.1]	[71.2;80.8]		
Sex (F/M,%)	39.5/60.5	41.7/58.3	37.3/62.7	Chi^2^ test	0.453
Body weight pre-dialysis^‡^ (kg)	70.9	71.9	70	Wilcoxon test	0.756
Median [Q1_Q3]	[61.3;80.6]	[61.2;79.4]	[61.7;81.0]		
Body weight post-dialysis^‡^ (kg)	68.7	69.8	68.2	Wilcoxon test	0.787
Median [Q1_Q3]	[59.9;78.4]	[59.6;77.7]	[60.0;79.3]		
Diabetes Mellitus (%)	39	36.6	41.4	Chi^2^ test	0.408
Hypertension (%)	76.6	71.8	81.4	Chi^2^ test	0.057
Congestive Heart Failure (%)	14.9	16.2	13.6	Chi^2^ test	0.536
Ischemic Cardiac Disease (%)	35.8	33.8	37.9	Chi^2^ test	0.478
Arrhythmia (%)	12.1	12	12.1	Chi^2^ test	0.965
Valvulopathy (%)	2.8	3.5	2.1	Fisher exact test	0.723
Aortic Disease (%)	2.5	2.8	2.1	Fisher exact test	0.999
Gut Ischemic Disease (%)	0.7	0.7	0.7	Fisher exact test	0.999
Limb Arteriopathy (%)	0.35	0.7	0	Fisher exact test	0.999
Ischemic CNS (%)	6.7	7.8	5.7	Chi^2^ test	0.496

Table 2 outlines the dialysis prescription and effective treatment delivered over the two-year follow-up period. Across the entire cohort, the median session time was 240 min based on thrice-weekly treatments. The median blood flow was 336.7 ml/min with a dialysate flow of 500 ml/min, and average weight loss of 2.1 kg per session, corresponding to a net ultrafiltration rate (NET QUF) of 8.7 ml/min. All patients were treated using single-use high-flux synthetic dialyzers with a median surface area of 1.8 m^2^. The choice of dialyzer was left to the discretion of the prescribing physician, and brand names were not collected for confidentiality reasons. Compared to HD, the HDF group had significantly higher blood flow (345.0 ml/min vs. 325.8 ml/min; p = 0.038) and achieved substitution and total ultrafiltration flows [Refer to Appendix for Complementary Information] of 89.0 and 97.7 ml/min, respectively, resulting in a median total ultrafiltration volume of 23.2 L per HDF session.

**Table 2:** Dialysis prescription and delivered treatment over two years, showing similar session parameters across groups, with higher blood flow and ultrafiltration volumes in the HDF group.

Characteristics	Total	HD	HDF	Test	p-value
Patients	286	144	142		
Treatment Time (t_HD_) (min) ^‡^ Median [Q1-Q3]	240.0 [236.7; 240.0]	240.0 [236.7.0;240.0]	240.0 [237.3;240.0]	Wilcoxon test	0.945
QB (ml/min) Median [Q1-Q3]	336.7 [301.0; 366.7]	325.8 [300.0; 365.0]	345.0 [313.3; 366.7]	Wilcoxon test	0.038
QD (ml/min) Median [Q1-Q3]	500 [500; 500]	500 [500; 500]	500 [500; 500]		
QSUB (ml/min) Median [Q1-Q3]	89.0 [79.0;103.5]	NA	89.0 [79.0;103.5]		
TOTAL QUF (ml/min) Median [Q1-Q3]	14.7 [8.8;97.7]	8.8 [7.2;10.8]	97.7 [86.7;110.9]	Wilcoxon test	< 0.001
Weight Loss (Kg) Median [Q1-Q3]	2.1 [1.6;2.6]	2.1 [1.6;2.6]	2.1 [1.6;2.6]	Wilcoxon test	0.881
NET QUF (ml/min) Median [Q1-Q3]	8.7 [7.2;10.9]	8.8 [7.2;10.8]	8.7 [7.1;11.1]	Wilcoxon test	0.973
Total^‡^ Ultrafiltration Vol (L/ses) Median [Q1-Q3]	3.3 [2.0;23.2]	2.0 [1.6;2.6]	23.2 [19.4;26.0]	Wilcoxon test	< 0.001

Table 3 presents key metrics of dialysis efficiency and adequacy. HDF demonstrated superior solute clearance compared to high-flux HD. The reduction ratio for small solute urea was higher in the HDF group (78.5% vs. 75.9%; p = 0.008), along with greater single-pool Kt/V (1.79 vs. 1.66; p = 0.009) and total Kt (64.8 vs. 62.4 l/session; p = 0.013). Middle molecule clearance using ß2M-related parameters also favored HDF. The ß2M reduction ratio was significantly higher in the HDF group (69.6% vs.58.7%; p < 0.001) as was the effective ß2M clearance (59.3 vs. 44.1 ml/min; p < 0.001) and ß2M mass removal (208.5 vs 183.2 mg/session; p = 0.002), resulting in a lower ß2M time averaged concentration (β2M TAC: 17.3 vs. 15.1; p < 0.001). Nutritional parameters, including normalized protein catabolic rate (nPCR, g/kg/24h) and serum albumin, were comparable between groups. In terms of middle molecule clearance, HDF significantly outperformed HD, achieving a ß2M reduction ratio of 69.6% versus 58.7%, along with superior clearance and mass removal, resulting in lower ß2M plasma concentrations.

**Table 3:** Dialysis efficiency and adequacy metrics, highlighting superior small and middle molecule clearance with HDF, while nutritional parameters remained comparable between groups.

Parameters	Total	HD	HDF	Test	p-value
Patients (n)	286	144	142		
PR Urea (%) Median [Q1_Q3]	77.2 [73.3 ; 81.1]	75.9 [72.1 ; 80.3]	78.5 [74.9 ; 81.3]	Wilcoxon test	0.008
Kt/V^‡^ Median [Q1_Q3]	1.7 [1.5 ; 1.9]	1.7 [1.5 ; 1.9]	1.8 [1.6 ; 2.0]	Wilcoxon test	0.009
Kt (L/ses) ^‡^ Median [Q1_Q3]	63.1 [55.2 ; 71.8]	62.4 [52.0 ; 69.3]	64.8 [57.1 ; 75.7]	Wilcoxon test	0.013
nPCR (g/kg/24 h) Median [Q1_Q3]	1.1 [1.0 ; 1.3]	1.1 [1.0 ; 1.2]	1.1 [0.9 ; 1.3]	Wilcoxon test	0.946
ß2M_pre_ (mg/l) ^‡^ Median [Q1_Q3]	26.2 [22.9 ; 30.5]	26.9 [23.4 ; 30.9]	26.0 [22.5 ; 29.9]	Wilcoxon test	0.122
ß2M_post.eq_ (mg/l) ^‡^ Median [Q1_Q3]	9.3 [7.3 ; 11.9]	10.9 [9.0 ; 13.9]	8.1 [6.6 ; 10.2]	Wilcoxon test	< 0.001
PR ß2M (%) Median [Q1_Q3]	63.2 [55.9 ; 71.1]	58.7 [51.7 ; 63.8]	69.6 [62.2 ; 73.0]	Wilcoxon test	< 0.001
ß2M _TAC_ (mg/l) ^‡^ Median [Q1_Q3]	16.3 [13.9 ; 19.1]	17.3 [15.3 ; 20.5]	15.1 [12.9 ; 17.9]	Wilcoxon test	< 0.001
Effective K_ß2M_ (ml/min) Median [Q1_Q3]	52.5 [41.9 ; 61.9]	44.1 [38.0 ; 54.7]	59.3 [50.2 ; 68.5]	Wilcoxon test	< 0.001
ß2M Mass removal (mg/ses) Median [Q1_Q3]	197.5 [157.5 ; 240.9]	183.2 [142.3 ; 219.3]	209.5 [169.2 ; 253.6]	Wilcoxon test	0.002

This section reports treatment efficiency as assessed by the estimated continuous dialytic clearance of ß2M (eCDC_ß2M_) and explores its association with the convection volume delivered during dialysis.

**Fig. 1:**
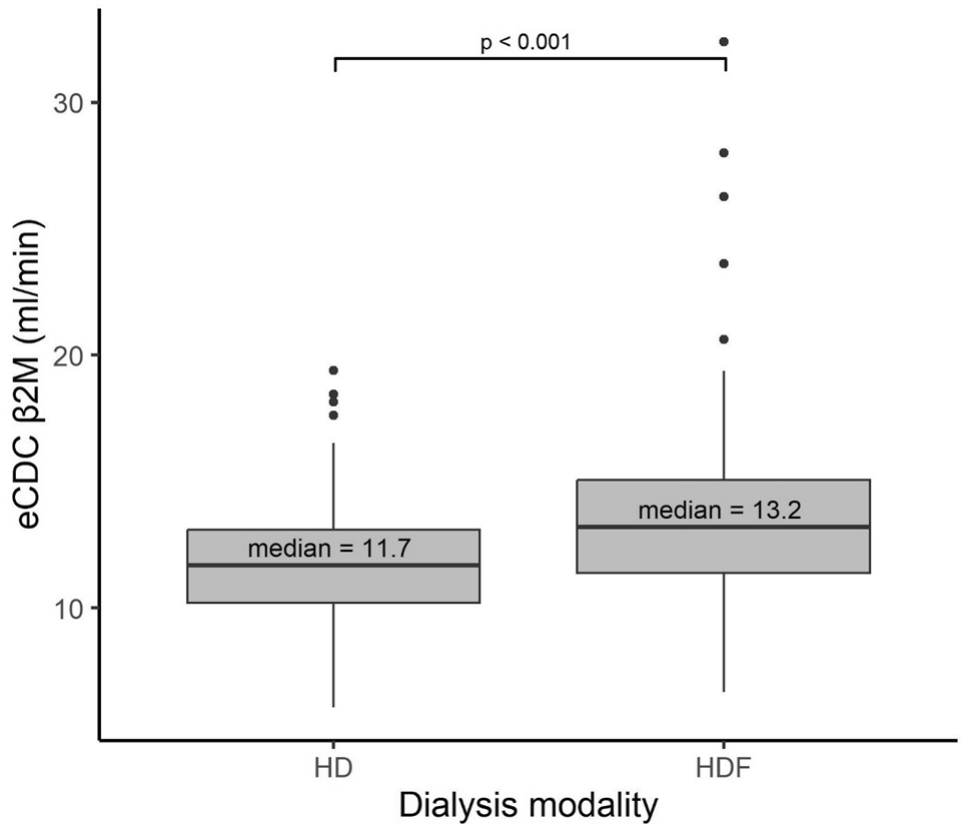
Median eCDC_ß2M_ values over two years by treatment modality, showing significantly higher effective ß2M clearance with HDF compared to HD.

Figure 1 shows eCDC_ß2M_ values stratified by treatment modality presented as medians with interquartile ranges over a two-year period. Patients treated with HDF exhibited significantly higher eCDC_β2M_ values compared to those on HD, with a median of 13.2 ml/min versus 11.7 ml/min (p < 0.001), suggesting that HDF provided an additional 1.5 ml/min of effective ß2M clearance under comparable clinical conditions.

**Fig. 2:**
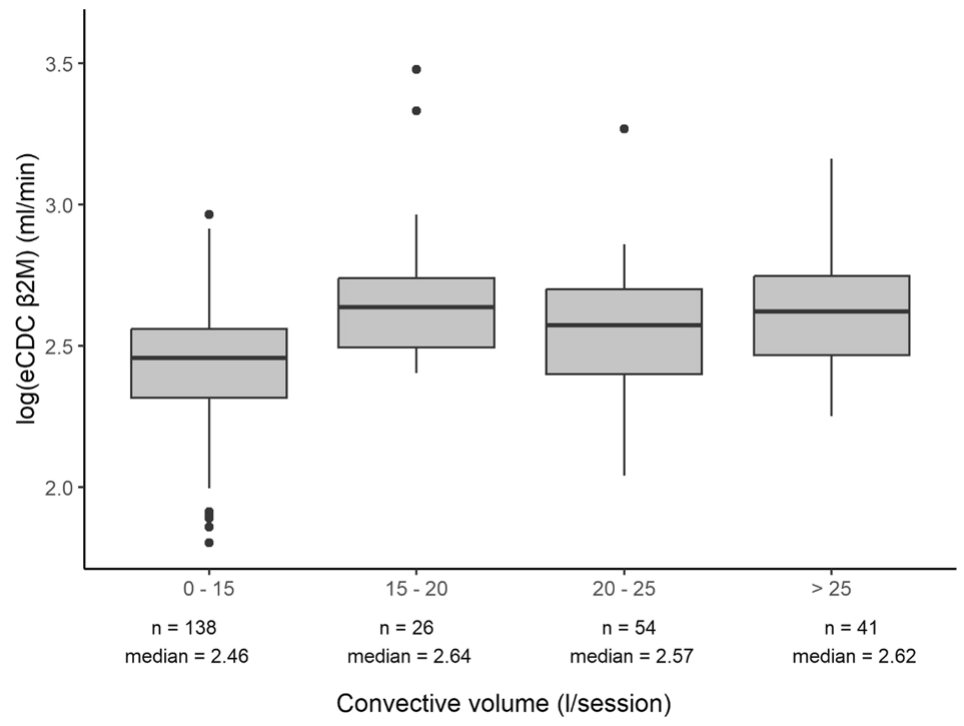
Log-transformed eCDC_ß2M_ values by convective volume class, showing a significant linear relationship between higher convection volumes and increased middle molecule clearance.

Figure 2 shows box plots of log-transformed eCDC_β2M_ values plotted against convective volume (CV) per session, grouped into four predefined CV groups across the entire cohort. The relationship between CV and eCDC_β2M_ can be estimated using the equation log (eCDC_β2M_) = 2.42 + 0.0007 CV (p < 0.0001), indicating a statistically significant linear increase in log(eCDC_β2M_) with increasing CV. The first CV group (0–15 L/session) corresponds to patients whose convective volume reflects net ultrafiltration aimed solely at fluid removal. Median log(eCDC_β2M_) values for each group, using eCDC_β2M_ values averaged over two years, demonstrated a consistent upward trend with increasing CV, highlighting the positive impact of higher convection volumes on middle molecule clearance efficiency.

This section examines the prognostic value of the estimated continuous dialytic clearance of ß2M (eCDC_ß2M_) on both all-cause and cardiovascular mortality.

**Fig. 3:**
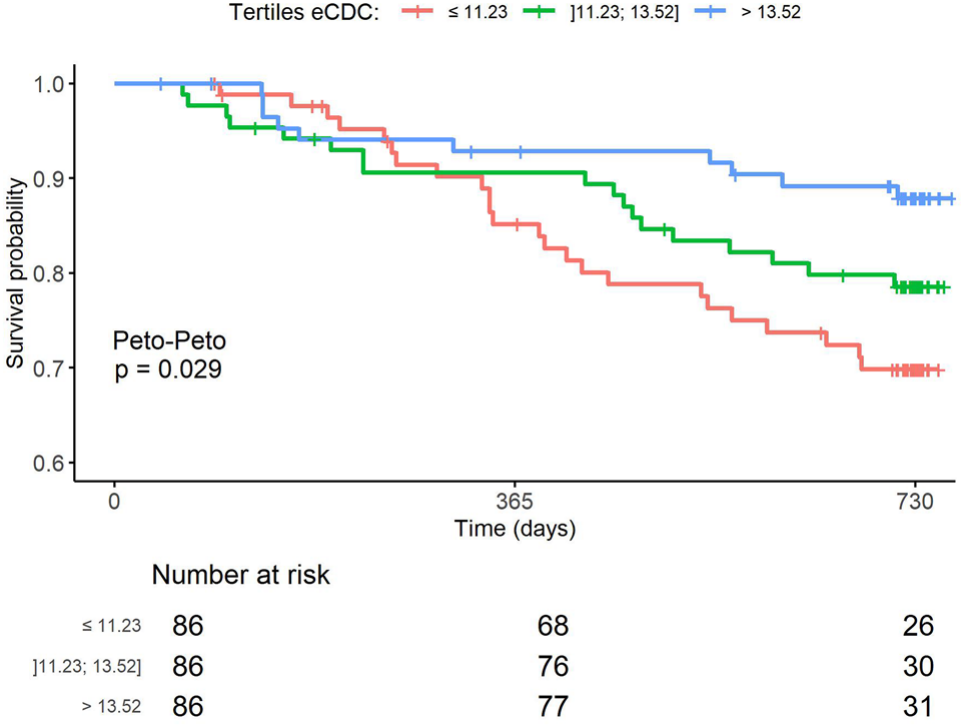
All-cause 2-year survival by eCDC_ß2M_ tertiles, showing significantly better survival in patients with effective ß2M clearance above 13.52 ml/min.

Figure 3 illustrates all-cause survival probability stratified by tertiles of eCDC_β2M_ for the combined cohort of HD and HDF patients. Patients in the highest tertile (> 13.52 ml/min) demonstrated significantly greater 2-year survival compared to those in the middle (11.23–13.52 ml/min) and lowest (≤ 11.23 ml/min) tertiles (p = 0.021). These findings suggest that a higher delivered dialysis dose, as reflected by eCDC_β2M_ above 13.52 ml/min, is associated with a meaningful survival benefit.

**Fig. 4:**
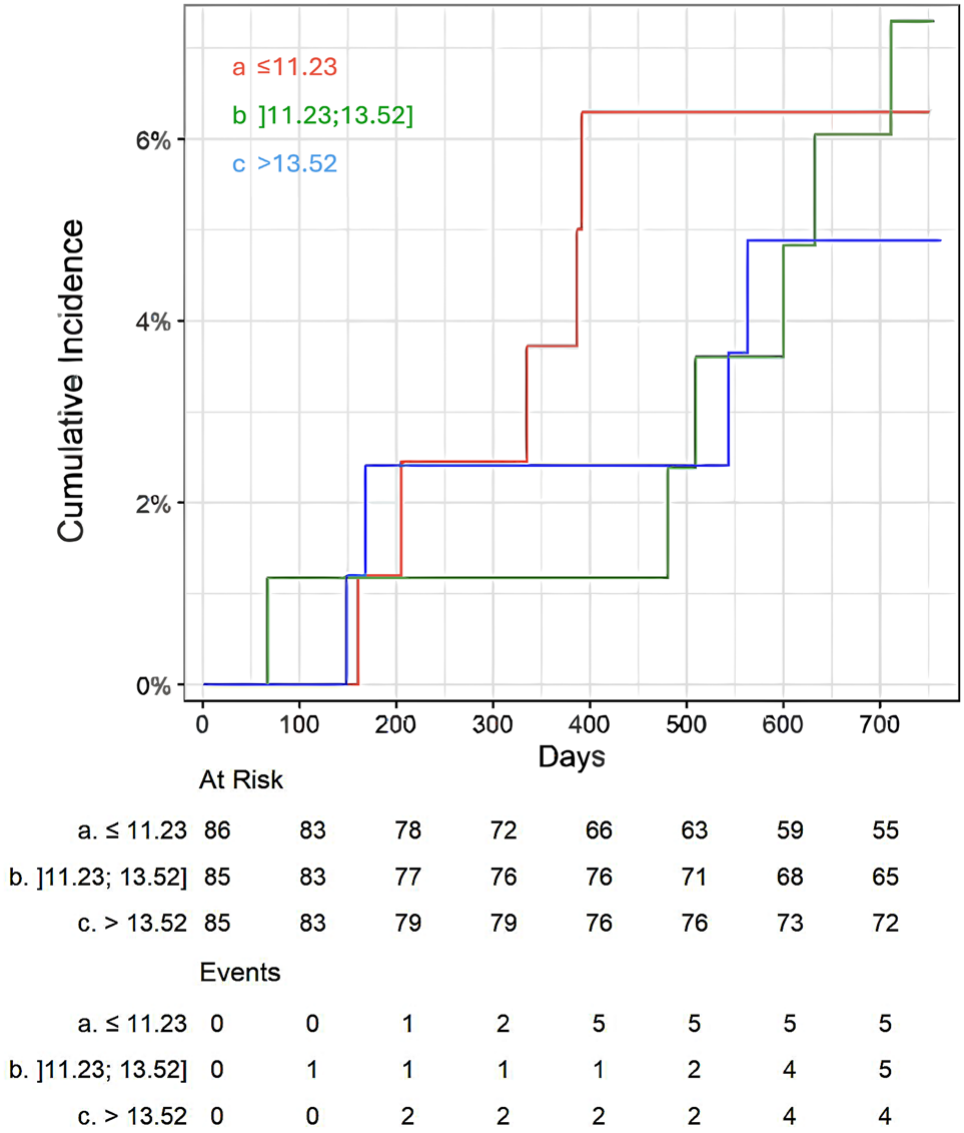
Cumulative incidence of cardiovascular mortality by eCDC_ß2M_ tertiles, showing no significant differences across groups.

Figure 4 presents the cumulative incidence of cardio-vascular mortality for the same eCDC_β2M_ tertiles. In contrast to all-cause mortality, no statistically significant difference was observed between tertiles (p = 0.83), indicating that eCDCß2M may not independently predict cardiovascular mortality in this population.

This section reports the comparative predictive effect of eCDC_ß2M_ and urea-based Kt/V on the risk of all-cause mortality in the overall patient cohort.

Table 4 shows the results of Cox proportional hazard (PH) models evaluating the association of eCDC_ß2M_ and urea Kt/V with all-cause mortality in both HD and HDF patients. Higher eCDC_β2M_ values were significantly associated with a reduced mortality risk (HR 0.81; 95% CI [0.72; 0.92]), indicating a protective effect of greater middle molecule clearance. In contrast, urea-based Kt/V was not significantly associated with mortality (HR 0.81; 95% CI [0.28; 2.34]). Among clinical covariates, older age, female sex, diabetes, hypertension, and heart failure did not show a significant impact on mortality risk. However, the presence of ischemic heart disease was associated with an increased risk of death (HR 1.9; 95% CI [1.10; 3.40]). Notably, CRP levels did not significantly influence mortality when modeled alongside eCDC_ß2M_.

**Table 4:** Cox regression analysis of all-cause mortality, showing a significant protective effect of higher eCDC_ß2M_, while urea Kt/V and most clinical covariates were not associated with mortality risk.

Parameter	Comparison	HR [95% CI]	p-value
eCDC _ß2M_		0.8138 [0.7214 ; 0.918]	< .001
Kt/V		0.8066 [0.2774 ; 2.345]	0.692
Age	≥ 76.2 yo vs. < 76.2 yo	1.4130 [0.7976 ; 2.503]	0.233
Sex	Male vs. Female	0.8537 [0.4324 ; 1.686]	0.650
Diabetes	Yes vs. No	1.0904 [0.5987 ; 1.986]	0.778
Hypertension	Yes vs. No	1.9220 [0.8823 ; 4.187]	0.080
Heart failure	Yes vs. No	1.1333 [0.5426 ; 2.367]	0.742
Ischemic cardiac disease	Yes vs. No	1.9015 [1.0648 ; 3.396]	0.031
Arrhythmia	Yes vs. No	0.7759 [0.3047 ; 1.976]	0.583
CRP		1.0079 [0.9935 ; 1.022]	0.311

Table 5 shows a multivariate Cox PH model after covariable selection, focusing on the predictive value of eCDC_ß2M_ for all-cause mortality. Consistent with the previous model, eCDC_β2M_ remained a strong and independent predictor of survival (HR 0.82; 95%CI [0.74; 0.92)] with higher values associated with a greater protective effect. In this refined model, ischemic cardiac disease continued to be significantly associated with increased mortality (HR 2.04; 95% CI [1.20; 3.60]).

**Table 5:** Refined multivariable Cox model confirming eCDC_ß2M_ as an independent predictor of improved survival, while ischemic cardiac disease remained significantly associated with higher mortality.

Parameter	Comparison	HR [95% CI]	p-value
eCDC _ß2M_		0.8226 [0.7373 ; 0.9223]	< .001
Ischemic Cardiac Disease	Yes vs. No	2.0367 [1.1629 ; 3.5670]	0.014

Table 6 presents a breakdown of mortality and hospitalization by cause, expressed as events per 100 patient-years, extrapolated from the original study report. As shown, there is a trend toward lower mortality with HDF compared to HD 11.51 vs. 13.82 for all-cause mortality and 21.74 vs. 26.35 for cardiac-related deaths. However, these differences, including those related to hospitalization causes, did not reach statistical significance.

**Table 6:** All-Cause and Cardiovascular Mortality, Including Cause-Specific Cardiovascular Mortality, Reported in the FRENCHIE Study.

Mortality	HD (Events/100 pt-yrs)	HDF (Events/100 pt-yrs)	*p-value*
All-Cause Mortality	13.82	11.51	0.43
Cardiovascular Death	26.35	21.74	0.53
Sudden Cardiac Death	2.89	2.24	ns
Hospitalization			
Heart Failure	3.86	2.24	ns
Ischemic Heart Disease	9.64	8.31	ns
Stroke	4.82	2.88	ns
Arrhythmia	5.14	6.07	ns

## Discussion

The original aim and findings of this study were to quantify and compare the efficiency of kidney replacement therapies (HD vs. HDF) using a biomarker reported to reflect glomerular filtration rate and providing a proxy for a continuous dialysis clearance, allowing for a comparison with native kidney function. For this purpose, we utilized plasma ß2M levels measured in patients enrolled in our previous FRENCHIE study^32^. All ß2M samples were measured in a central laboratory, so reducing or mitigating inter-laboratory variations.

As established by the CKD-EPI consortium and subsequently validated by Argyropoulos and colleagues, using a large dataset of patients collected through their meta-analyses spanning various stages of chronic kidney disease and utilizing advanced simulation modeling, plasma ß2M concentrations may be accurately correlated with measured glomerular filtration rates (GFR)^23,24,26^, with plasma ß2M concentrations displaying an exponential correlation with glomerular filtration rate. To address fluctuations in plasma ß2M levels from varying HD and HDF treatments, we calculated the time-averaged concentration of ß2M (ß2M_TAC_) over the mid-week dialysis session^40,41^ for each patient. This midweek ß2M_TAC_ was used to estimate the equivalent continuous dialytic clearance (eCDC_ß2M_) achieved by the two treatment modalities.

In brief, postdilution HDF with a medium convection volume averaging 23.2 L per session provides significantly higher efficiency across all molecular weight spectrums for small and middle molecules as represented by ß2M. As reported in our study, HDF yields a 12.7% lower β2M_TAC_ (15.1 mg/l) compared to HD (17.3 mg/l), and HDF yields a 12.8% higher eCDC_ß2M_ (13.2 ml/min) than high-flux HD (11.7 ml/min). Other indicators of dialysis efficiency for urea Kt/V, percent reduction of urea and β2M, as well as effective ß2M clearances (59.3 vs 44.1 ml/min) and β2M mass removal (209.5 vs 183.2 mg per session) confirmed the superiority of HDF compared to high-flux HD. Furthermore, the superior efficiency of HDF may be summarized in a + 1.5 ml/min eCDC_ß2M_ for the same treatment time and conditions compared to a standard 4-h HD-treatment. In addition, the data show that eCDC_ß2M_ increases as the total convection volume rises, reinforcing the survival benefits of delivering higher convective volumes to patients. However, the number of sessions achieving high convective volumes (> 25 L) was limited, so preventing further exploration of the impact of these higher volumes on eCDC_ß2M_.

Interestingly, survival expectancy, as calculated by Kaplan Meyer analysis across the entire population and stratified by tertiles of eCDC_ß2M_, confirmed the survival advantage of higher eCDC_ß2M_ values (> 13.52 ml/min) compared to the lower tertiles. The higher predictive and protective value of eCDC_ß2M_, as compared to Kt/V, was further supported by Cox regression multivariate analysis, even after adjusting for confounding factors including advanced age, female sex, hypertension, cardiac ischemic disease, and inflammation.

Notably, the use of eCDC_ß2M_ offers a practical marker for assessing the overall kidney replacement therapy efficiency, including when applicable, the contribution of residual kidney function^42–45^. Several studies have consistently highlighted the significant impact of residual kidney function on lowering ß2M circulating levels and improving patient outcomes in a dose-dependent manner^46,47^. Furthermore, ß2M levels have been proposed as a useful tool to guide and tailor incremental dialysis regimens, whether peritoneal dialysis or hemodialysis^48–55^. By utilizing eCDC_β2M_, dialysis patient management can be simplified, as it eliminates the need for routine urine collection and the calculation of residual kidney function. This streamlines the process for clinicians and healthcare providers while reducing the burden on dialysis patients.

This proposed concept of eCDC_β2M_ should be integrated into the ongoing search for more suitable markers of dialysis efficiency, alongside traditional urea fractional clearance (Kt/V), particularly when considering convective-based therapies^43^. In this context, the metabolic, pharmacokinetic, and toxic properties of ß2M make it an ideal biomarker for this purpose. ß2M reflects kidney function, inflammation, cell activation, and the risk of poor outcomes associated with uremic toxin exposure^24,26,56,57^. Our approach and findings suggest that eCDC_ß2M_ may offer a novel metric for assessing dialysis efficiency, one that is more closely aligned with the native glomerular filtration rate (GFR). Additionally, it provides a means to compare the efficiency of various treatment regimens, from incremental to intensive dialysis, as well as conventional thrice-weekly sessions. This concept is in line with a previous proposal by Casino and co-workers, which proposed the use of a more appropriate metric than urea Kt/V to assess effective dialysis efficiency, advocating an equivalent of urea clearance as a proxy marker of a weekly continuous clearance^58,59^. Despite the interest in this original concept, the Casino formula never really gained popularity as it was solely based on urea, a poor marker of uremic toxicity, and an inadequate indicator of glomerular filtration rate^58,59^.

Using ß2M kinetic modeling analysis or percent reduction, it has been demonstrated that HDF provides a removal rate capacity up to 30% higher than high flux HD^39,60–62^. Additionally, the percent reduction or effective clearance of ß2M is positively correlated with convection volume^63,64^. The superior efficiency of HDF has been confirmed using a global removal score approach that uses markers across a broad molecular weight spectrum, ranging from 60 Daltons to 41 kDa, and adjusted for albumin loss^65,66^. Such superior removal rate translates into higher mass removal that tends to reduce ß2M circulating levels over time. In this context, a post-hoc analysis of the HEMO study showed that predialysis ß2M concentrations represented a biomarker for an increased risk of mortality for HD patients, with a threshold value of 27 mg/l^67,68^. Interestingly, this relative risk increased up to 60% when ß2M levels reached 50 mg/l. The membrane permeability outcome (MPO) study subsequently showed in its primary outcome that high-flux HD improved survival expectancy of diabetic and hypoalbuminemic HD patients, suggesting a benefit of clearing middle molecular weight uremic compounds^69^. Unfortunately, ß2M concentrations were not reported in the MPO study. Based on these findings, the European Best Practices Guidelines pointed out the need for clearing and assessing middle molecule uremic toxins with a particular focus on ß2M. In addition, the Japanese Society for Dialysis Therapy included ß2M concentrations as a marker of HDF efficiency in their own best practice guidelines, setting pre-dialysis target levels of ß2M of 25 mg/l for HDF and 27 mg/l for high flux HD^70^.

Recent update reviews have confirmed the increasing interest in monitoring ß2M in chronic kidney disease patients for assessing both kidney function and mortality risk. This interest in ß2M is not only limited to dialysis, but also to other specialties including cardiology when assessing the risk of mortality following an acute ischemic cardiac event, confirming the predictive value of this biomarker as a proxy of endothelial dysfunction and more broadly vascular damage. Furthermore, ß2M kinetic modeling has recently been revisited by Ward et al. in an interesting approach based on real-world data^71^. They provided simulated clearances of ß2M and projected levels of time-averaged concentration of ß2M according to treatment modalities, comparing high flux HD and HDF, relying on various convection doses and sieving properties of membranes. Their kinetic modeling showed that the percent reduction of ß2M increased parabolically with increased substitution volume in a dose-dependent manner, consequently leading to a decrease in ß2M_TAC_. Additionally, using the CONVINCE simulated data, they forecasted that high-volume HDF would have decreased ß2M_TAC_ to around 14 mg/l, compared to 16 mg/l in high-flux HD relying on the 26 L convection volume used, given that patients generate up to 240 mg of ß2M daily. Interestingly, their predictions for ß2M concentrations closely align with our current findings.

The strength of this study lies in the novel development of a new metric for dialysis efficiency closely aligned with native glomerular filtration rate, and by providing a precise method to compare dialysis modalities. As shown, in this analysis, HDF conducted within the same timeframe and virtually identical operating conditions, with a median convection volume of 23 L yielded a + 1.5ml/min equivalent glomerular filtration rate compared to high flux HD. This finding may align with previous studies that have emphasized the significant importance of residual kidney function on clinical benefits and improved patient outcomes. As indicated, survival benefits rely on an eGFR greater than 13.2 ml/min which can be achieved with a convection volume of 23 L per session of. Additionally, it has been demonstrated that increasing the convection volume to more than 25 L provides additional benefits, as it is associated with a higher eCDC. It can then be speculated that the 26,5 L of convection volume delivered in the CONVINCE study will correspond to about 16 ml/min and this may explain the survival benefit observed. Such an eGFR value may reflect the upper convective doses delivered in the ESHOL study, so explaining their reported clinical benefits.

The limitations of this study must be acknowledged. Firstly, it is a post hoc analysis of the FRENCHIE study, where the intended convective dose of 24 L per session was not uniformly achieved, potentially impacting the ability to show improved survival with HDF in the elderly population. Second, the narrow range of convective doses delivered constrained the precision of eCDC_ß2M_ estimates at higher convection volumes, particularly beyond 25 L per session. Third, the relatively limited number of cardiac events observed (as shown in Table 6) limited the statistical power to assess the relationship between eCDCß2M and cardiovascular mortality or hospitalization, precluding any definitive conclusion on this outcome. Fourth, plasma ß2M concentrations can be influenced by factors beyond dialysis efficiency and residual kidney function, such as infections and systemic inflammation. For this reason, regular ß2M measurements are recommended to establish a stable baseline and accurately assess treatment efficacy. Nevertheless, in our study, CRP levels did not suggest significant inflammatory activity, as reflected by their stable distribution across the patient quartiles. Fifth, residual kidney function was not factored into the analysis, as it was considered negligible due to the advanced dialysis vintage (mean 5 years) in this cohort. Nevertheless, it is important to note that in patients with preserved residual function, additional native ß2M clearance would likely contribute to higher eCDCß2M values and potentially better outcomes.

From a clinician’s perspective, as developed in this study, the use of eCDC_ß2M_ offers a novel integrated and goal-directed approach to assess effective dialysis efficiency across dialysis modalities, considering native glomerular filtration rate as reference, with prognostic value for patient outcomes. However, further and larger studies are necessary to validate the proposed concept of estimating eCDC_ß2M_ from circulating levels of ß2M in end-stage chronic kidney disease patients on maintenance dialysis. Additionally, comparing the effective eCDC_ß2M_ delivered in intensive dialysis such as nocturnal, alternate day, or daily treatment programs would be essential to link their better outcomes to some tangible biomarker. Despite its limitations, eCDC_ß2M_ may already provide a relatively simple and reliable metric to assess the treatment efficiency across all modalities of kidney replacement therapies, including the potential effect of residual kidney function.

## Conclusion

ß2M-based eCDC provides a new tool that facilitates the comparison of dialysis efficiency across different modalities, incorporating residual kidney function. As demonstrated in our study, HDF offers higher treatment efficiency compared to high-flux HD, which can be quantified as an increase of + 1.5 ml/min in eCDC_ß2M_ for a substitution volume of 23 L. This novel approach aligns more closely with native kidney function, enhancing and allowing personalizing patient management as part of a goal-oriented strategy, and potentially improving patient outcomes. However, this concept requires further validation and exploration of its predictive value across diverse dialysis modalities and patient cohorts.

## Supplementary Information

Supplementary Information.

## Data Availability

The data supporting the findings of this study are not publicly available due to privacy concerns that could compromise the confidentiality of research participants. However, the data can be obtained from the corresponding author [BC] upon reasonable request, subject to appropriate ethical and privacy considerations.

## References

[1] Peters, S. A. et al. Haemodiafiltration and mortality in end-stage kidney disease patients: a pooled individual participant data analysis from four randomized controlled trials. *Nephrol. Dial Transplant.* **31**(6), 978–984 (2016). 26492924 10.1093/ndt/gfv349

[2] Davenport, A. et al. Higher convection volume exchange with online hemodiafiltration is associated with survival advantage for dialysis patients: the effect of adjustment for body size. *Kidney Int.* **89**(1), 193–199 (2016). 26352299 10.1038/ki.2015.264

[3] Blankestijn, P. J. et al. Effect of Hemodiafiltration or Hemodialysis on Mortality in Kidney Failure. *N. Engl. J. Med.* **389**(8), 700–709 (2023). 37326323 10.1056/NEJMoa2304820

[4] Maduell, F. et al. High-efficiency postdilution online hemodiafiltration reduces all-cause mortality in hemodialysis patients. *J. Am. Soc. Nephrol.* **24**(3), 487–497 (2013). 23411788 10.1681/ASN.2012080875 PMC3582206

[5] Maduell, F. et al. Hemodiafiltration Reduces All-Cause and Cardiovascular Mortality in Incident Hemodialysis Patients: A Propensity-Matched Cohort Study. *Am. J. Nephrol.* **46**(4), 288–297 (2017). 29041011 10.1159/000481669

[6] Canaud B, Blankestijn PJ, Grooteman MPC, Davenport A. Why and how high volume hemodiafiltration may reduce cardiovascular mortality in stage 5 chronic kidney disease dialysis patients? A comprehensive literature review on mechanisms involved. Semin Dial. (2021). 10.1111/sdi.13039 34842306

[7] Daugirdas JT, Chan CT. Survival Benefit with Hemodiafiltration: Are We Convinced, and If So, What Might Be the Mechanism? Clin. J. Am. Soc. Nephrol. (2023). 10.2215/CJN.0000000000000355 PMC10937017 37902765

[8] Carney, E. F. CONVINCE trial reports a survival benefit of haemodiafiltration compared with haemodialysis. *Nat. Rev. Nephrol.* **19**(8), 478 (2023). 37365327 10.1038/s41581-023-00740-x

[9] Mayne, K. J. & Ronco, C. Will another trial CONVINCE nephrologists to adopt high-dose haemodiafiltration over conventional haemodialysis?. *Clin. Kidney J.* **16**(12), 2393–2395 (2023). 38046007 10.1093/ckj/sfad258 PMC10689160

[10] Meena P, Locatelli F. Unmasking the CONVINCE trial: is hemodiafiltration ready to steal the spotlight in real-world practice? Clin. Kidney J. (2024). 10.1093/ckj/sfad247 PMC10768775 38186893

[11] Daugirdas, J. T. et al. Standard Kt/Vurea: a method of calculation that includes effects of fluid removal and residual kidney clearance. *Kidney Int.* **77**(7), 637–644 (2010). 20107428 10.1038/ki.2009.525

[12] Daugirdas JT, Leypoldt JK, Akonur A, Greene T, Depner TA, Group FHNT. 2013 Improved equation for estimating single-pool Kt/V at higher dialysis frequencies. Nephrol. Dial Transplant.. (2013). 10.1093/ndt/gfs115 PMC3765020 22561585

[13] Greene, T. et al. Association of achieved dialysis dose with mortality in the hemodialysis study: an example of “dose-targeting bias”. *J. Am. Soc. Nephrol.* **16**(11), 3371–3380 (2005). 16192421 10.1681/ASN.2005030321

[14] Bowry, S. K. & Canaud, B. Achieving high convective volumes in on-line hemodiafiltration. *Blood Purif.* **35**(Suppl 1), 23–28 (2013). 23466374 10.1159/000346379

[15] Canaud, B. et al. Optimal convection volume for improving patient outcomes in an international incident dialysis cohort treated with online hemodiafiltration. *Kidney Int.* **88**(5), 1108–1116 (2015). 25945407 10.1038/ki.2015.139 PMC4653588

[16] Canaud, B. & Bowry, S. K. Emerging clinical evidence on online hemodiafiltration: does volume of ultrafiltration matter?. *Blood Purif.* **35**(1–3), 55–62 (2013). 23343547 10.1159/000345175

[17] Canaud, B., Koehler, K., Bowry, S. & Stuard, S. What Is the Optimal Target Convective Volume in On-Line Hemodiafiltration Therapy?. *Contrib. Nephrol.* **189**, 9–16 (2017). 27951545 10.1159/000450634

[18] Chapdelaine I, de Roij van Zuijdewijn CL, Mostovaya IM, Lévesque R, Davenport A, Blankestijn PJ, et al. Optimization of the convection volume in online post-dilution haemodiafiltration: practical and technical issues. Clin. Kidney J. (2015). 10.1093/ckj/sfv003 PMC4370303 25815176

[19] Maduell, F. Optimizing the prescription of hemodiafiltration. *Contrib. Nephrol.* **158**, 225–231 (2007). 17684362 10.1159/000107254

[20] Maduell, F. Is There an “Optimal Dose” of Hemodiafiltration?. *Blood Purif.* **40**(Suppl 1), 17–23 (2015). 26344509 10.1159/000437409

[21] Jaffrin, M. Y. Convective mass transfer in hemodialysis. *Artif. Organs.* **19**(11), 1162–1171 (1995). 8579528 10.1111/j.1525-1594.1995.tb02277.x

[22] Jaffrin, M. Y., Ding, L. H. & Laurent, J. M. Simultaneous convective and diffusive mass transfers in a hemodialyser. *J. Biomech. Eng.* **112**(2), 212–219 (1990). 2345453 10.1115/1.2891174

[23] Argyropoulos, C. P. et al. Rediscovering Beta-2 Microglobulin As a Biomarker across the Spectrum of Kidney Diseases. *Front Med. (Lausanne).* **4**, 73 (2017). 28664159 10.3389/fmed.2017.00073 PMC5471312

[24] Roumelioti, M. E., Nolin, T., Unruh, M. L. & Argyropoulos, C. Revisiting the Middle Molecule Hypothesis of Uremic Toxicity: A Systematic Review of Beta 2 Microglobulin Population Kinetics and Large Scale Modeling of Hemodialysis Trials In Silico. *PLoS ONE* **11**(4), e0153157 (2016). 27055286 10.1371/journal.pone.0153157 PMC4824495

[25] Achakzai MI, Argyropoulos C, Roumelioti ME. Predicting Residual Function in Hemodialysis and Hemodiafiltration-A Population Kinetic, Decision Analytic Approach. J. Clin. Med. (2019). 10.3390/jcm8122080 PMC6947429 31795401

[26] Roumelioti, M. E. et al. Beta-2 microglobulin clearance in high-flux dialysis and convective dialysis modalities: a meta-analysis of published studies. *Nephrol. Dial Transplant.* **33**(6), 1025–1039 (2018). 29186592 10.1093/ndt/gfx311

[27] Liabeuf, S. et al. Plasma beta-2 microglobulin is associated with cardiovascular disease in uremic patients. *Kidney Int.* **82**(12), 1297–1303 (2012). 22895515 10.1038/ki.2012.301

[28] Shi, F., Sun, L. & Kaptoge, S. Association of beta-2-microglobulin and cardiovascular events and mortality: A systematic review and meta-analysis. *Atherosclerosis* **320**, 70–78 (2021). 33581388 10.1016/j.atherosclerosis.2021.01.018 PMC7955279

[29] Gong, S. et al. Elevated serum beta-2 microglobulin level predicts short-term poor prognosis of patients with de novo acute omicron variant COVID-19 infection. *Front Cell Infect Microbiol.* **13**, 1204326 (2023). 37520437 10.3389/fcimb.2023.1204326 PMC10373586

[30] Bethea, M. & Forman, D. T. Beta 2-microglobulin: its significance and clinical usefulness. *Ann. Clin. Lab Sci.* **20**(3), 163–168 (1990). 2188562

[31] Bataille R, Grenier J. Serum beta 2 microglobulin in multiple myeloma. A critical review. Eur J Cancer Clin. Oncol. (1987). 10.1016/0277-5379(87)90047-2 3325293

[32] Morena, M. et al. Treatment tolerance and patient-reported outcomes favor online hemodiafiltration compared to high-flux hemodialysis in the elderly. *Kidney Int.* **91**(6), 1495–1509 (2017). 28318624 10.1016/j.kint.2017.01.013

[33] Zhang, W. R. & Parikh, C. R. Biomarkers of Acute and Chronic Kidney Disease. *Annu. Rev. Physiol.* **81**, 309–333 (2019). 30742783 10.1146/annurev-physiol-020518-114605 PMC7879424

[34] Inker, L. A. et al. A New Panel-Estimated GFR, Including β(2)-Microglobulin and β-Trace Protein and Not Including Race, Developed in a Diverse Population. *Am. J. Kidney Dis.* **77**(5), 673–83.e1 (2021). 33301877 10.1053/j.ajkd.2020.11.005 PMC8102017

[35] Ward, R. A. & Daugirdas, J. T. Kinetics of β -2-Microglobulin with Hemodiafiltration and High-Flux Hemodialysis. *Clin. J. Am. Soc. Nephrol.* **19**(7), 869–876 (2024). 38650079 10.2215/CJN.0000000000000461 PMC11254023

[36] Bergström, J. & Wehle, B. No change in corrected beta 2-microglobulin concentration after cuprophane haemodialysis. *Lancet* **1**(8533), 628–629 (1987). 2881162 10.1016/s0140-6736(87)90266-2

[37] Tattersall, J. Clearance of beta-2-microglobulin and middle molecules in haemodiafiltration. *Contrib. Nephrol.* **158**, 201–209 (2007). 17684359 10.1159/000107251

[38] Tattersall, J. E. & Ward, R. A. Online haemodiafiltration: definition, dose quantification and safety revisited. *Nephrol. Dial Transplant.* **28**(3), 542–550 (2013). 23345621 10.1093/ndt/gfs530

[39] Ward, R. A., Greene, T., Hartmann, B. & Samtleben, W. Resistance to intercompartmental mass transfer limits beta2-microglobulin removal by post-dilution hemodiafiltration. *Kidney Int.* **69**(8), 1431–1437 (2006). 16395268 10.1038/sj.ki.5000048

[40] Paats, J. et al. Time-averaged concentration estimation of uraemic toxins with different removal kinetics: a novel approach based on intradialytic spent dialysate measurements. *Clin. Kidney J.* **16**(4), 735–744 (2023). 37007697 10.1093/ckj/sfac273 PMC10061434

[41] Lopot, F., Nejedly, B. & Sulkova, S. Physiology in daily hemodialysis in terms of the time average concentration/time average deviation concept. *Hemodial Int.* **8**(1), 39–44 (2004). 19379400 10.1111/j.1492-7535.2004.00073.x

[42] Castro, I. & Rodrigues, A. Estimating Residual Kidney Function: Present and Future Challenge. *SN Comprehensive Clin. Med.* **2**(2), 140–148 (2020).

[43] Murea, M., Deira, J., Kalantar-Zadeh, K., Casino, F. G. & Basile, C. The spectrum of kidney dysfunction requiring chronic dialysis therapy: Implications for clinical practice and future clinical trials. *Semin. Dial.* **35**(2), 107–116 (2022). 34643003 10.1111/sdi.13027

[44] Shafi, T. & Levey, A. S. Measurement and Estimation of Residual Kidney Function in Patients on Dialysis. *Adv. Chronic Kidney Dis.* **25**(1), 93–104 (2018). 29499893 10.1053/j.ackd.2017.09.001 PMC5841591

[45] Steubl, D. et al. Development and Validation of Residual Kidney Function Estimating Equations in Dialysis Patients. *Kidney Med.* **1**(3), 104–114 (2019). 32734191 10.1016/j.xkme.2019.04.002 PMC7380427

[46] Okazaki, M. et al. Residual Kidney Function and Cause-Specific Mortality Among Incident Hemodialysis Patients. *Kidney Int. Rep.* **8**(10), 1989–2000 (2023). 37849997 10.1016/j.ekir.2023.07.020 PMC10577493

[47] Daugirdas, J. T. Residual Kidney Function and Cause-Specific Mortality. *Kidney Int. Rep.* **8**(10), 1914–1916 (2023). 37850019 10.1016/j.ekir.2023.08.024 PMC10577485

[48] Vilar, E. et al. Plasma Levels of Middle Molecules to Estimate Residual Kidney Function in Haemodialysis without Urine Collection. *PLoS ONE* **10**(12), e0143813 (2015). 26629900 10.1371/journal.pone.0143813 PMC4668015

[49] Vilar, E. & Farrington, K. Emerging importance of residual renal function in end-stage renal failure. *Semin. Dial.* **24**(5), 487–494 (2011). 21999737 10.1111/j.1525-139X.2011.00968.x

[50] Vilar, E. et al. Long-term outcomes in online hemodiafiltration and high-flux hemodialysis: a comparative analysis. *Clin. J. Am. Soc. Nephrol.* **4**(12), 1944–1953 (2009). 19820129 10.2215/CJN.05560809 PMC2798875

[51] Wong, J., Kaja Kamal, R. M., Vilar, E. & Farrington, K. Measuring Residual Renal Function in Hemodialysis Patients without Urine Collection. *Semin. Dial.* **30**(1), 39–49 (2017). 27757995 10.1111/sdi.12557

[52] Wong, J. et al. Predicting residual kidney function in hemodialysis patients using serum β-trace protein and β2-microglobulin. *Kidney Int.* **89**(5), 1090–1098 (2016). 26924065 10.1016/j.kint.2015.12.042

[53] Wong, J., Vilar, E., Davenport, A. & Farrington, K. Incremental haemodialysis. *Nephrol. Dial Transplant.* **30**(10), 1639–1648 (2015). 26038351 10.1093/ndt/gfv231

[54] Davenport, A. Will incremental hemodialysis preserve residual function and improve patient survival?. *Semin Dial.* **28**(1), 16–19 (2015). 25385441 10.1111/sdi.12320 PMC4320773

[55] Davenport, A. Measuring residual renal function for hemodialysis adequacy: Is there an easier option?. *Hemodial. Int.* **21**(Suppl 2), S41–S46 (2017). 29064172 10.1111/hdi.12592

[56] Canaud, B., Morena, M., Cristol, J. P. & Krieter, D. Beta2-microglobulin, a uremic toxin with a double meaning. *Kidney Int.* **69**(8), 1297–1299 (2006). 16612412 10.1038/sj.ki.5000389

[57] Maruyama, Y. et al. Association between serum beta2-microglobulin and mortality in Japanese peritoneal dialysis patients: A cohort study. *PLoS ONE* **17**(4), e0266882 (2022). 35421178 10.1371/journal.pone.0266882 PMC9009671

[58] Casino, F. G. & Lopez, T. The equivalent renal urea clearance: a new parameter to assess dialysis dose. *Nephrol. Dial Transplant.* **11**(8), 1574–1581 (1996). 8856214

[59] Casino, F. G. et al. A simple approach for assessing equilibrated Kt/V beta 2-M on a routine basis. *Nephrol. Dial Transplant.* **25**(9), 3038–3044 (2010). 20360013 10.1093/ndt/gfq173

[60] Leypoldt, J. K., Cheung, A. K. & Deeter, R. B. Rebound kinetics of beta2-microglobulin after hemodialysis. *Kidney Int.* **56**(4), 1571–1577 (1999). 10504510 10.1046/j.1523-1755.1999.00669.x

[61] Leypoldt, J. K., Holmes, C. J. & Rutherford, P. Clearance of middle molecules during haemodialysis and haemodiafiltration: new insights. *Nephrol. Dial Transplant.* **27**(12), 4245–4247 (2012). 23235952 10.1093/ndt/gfs475

[62] Leypoldt, J. K. et al. Intradialytic kinetics of middle molecules during hemodialysis and hemodiafiltration. *Nephrol. Dial Transplant.* **34**(5), 870–877 (2019). 30307514 10.1093/ndt/gfy304

[63] Lornoy, W., Becaus, I., Billiouw, J. M., Sierens, L. & van Malderen, P. Remarkable removal of beta-2-microglobulin by on-line hemodiafiltration. *Am. J. Nephrol.* **18**(2), 105–108 (1998). 9569951 10.1159/000013317

[64] Lornoy W, Becaus I, Billiouw JM, Sierens L, Van Malderen P, D'Haenens P. On-line haemodiafiltration. Remarkable removal of beta2-microglobulin. Long-term clinical observations. Nephrol. Dial Transplant. (2000). 10.1093/oxfordjournals.ndt.a027964 10737167

[65] Maduell, F. et al. Comparison of Solute Removal Properties Between High-Efficient Dialysis Modalities in Low Blood Flow Rate. *Ther. Apher. Dial.* **24**(4), 387–392 (2020). 31583845 10.1111/1744-9987.13440

[66] Maduell, F. et al. Medium Cut-Off Dialyzer versus Eight Hemodiafiltration Dialyzers: Comparison Using a Global Removal Score. *Blood Purif.* **48**(2), 167–174 (2019). 30943486 10.1159/000499759

[67] Cheung, A. K. et al. Serum beta-2 microglobulin levels predict mortality in dialysis patients: results of the HEMO study. *J. Am. Soc. Nephrol.* **17**(2), 546–555 (2006). 16382021 10.1681/ASN.2005020132

[68] Eknoyan, G. et al. Effect of dialysis dose and membrane flux in maintenance hemodialysis. *N. Engl. J. Med.* **347**(25), 2010–2019 (2002). 12490682 10.1056/NEJMoa021583

[69] Locatelli, F. et al. Effect of membrane permeability on survival of hemodialysis patients. *J. Am. Soc. Nephrol.* **20**(3), 645–654 (2009). 19092122 10.1681/ASN.2008060590 PMC2653681

[70] Watanabe, Y. et al. Japanese society for dialysis therapy clinical guideline for “hemodialysis initiation for maintenance hemodialysis”. *Ther. Apher. Dial.* **19**(Suppl 1), 93–107 (2015). 25817934 10.1111/1744-9987.12293

[71] Ward RA, Daugirdas JT. Kinetics of Beta2-Microglobulin with Hemodiafiltration and High-Flux Hemodialysis. Clin. J. Am. Soc. Nephrol. (2024). 10.2215/CJN.0000000000000461 PMC11254023 38650079

