# Supplementary material for: Continuous beta-2 microglobulin–based clearance highlights superiority of high-Dose HDF over high-flux HD in predicting outcomes
Source: Sci Rep. 2025 Jul 1;15:20421. doi: 10.1038/s41598-025-07497-2 (PMC12216211; doi:10.1038/s41598-025-07497-2)
Supplement: Supplementary file 1 — Supplementary Information. [file 41598_2025_7497_MOESM1_ESM.docx]

Appendix: Additional Explanations of Flow Definitions Used in HDF and Selected β2-Microglobulin Kinetic Parameters

- **Substitution, Ultrafiltration and Net Ultrafiltration Flows (38).**

Substitution flow (QSUB) is the infusion rate that compensate for total ultrafiltration (QUF) to maintain the patient’s isovolemic state. Total ultrafiltration flow (QUF) reflects the convective clearance. Net ultrafiltration flow (Net QUF) corresponds to the actual fluid removal (i.e., weight loss) and is calculated as QUF minus QSUB.

- **Pre- and post-dialysis ß2M concentrations.**

Pre- and post-dialysis ß2M concentrations were obtained from blood samples collected during midweek dialysis sessions. This timing was chosen to best reflect the steady-state equilibrium of the dialysis cycle, based on a conventional thrice-weekly treatment regimen.

- **Estimation of ß2M distribution volume.**

The distribution volume for ß2M was estimated as 20% of post-dialysis body weight for both male and female patients. This assumption reflects an isovolemic condition and is consistent with previous studies.

- **Time-averaged ß2M concentration.**

In the absence of pre-dialysis measurements from the next session, the logarithmic mean of the mid-week pre- and post-dialysis ß2M concentrations was used as the best estimate for the time-averaged concentration (TAC) across the dialysis cycle. This method has been validated in multiple studies, including one that used direct dialysate quantification based on UV absorbance measurements of dialysate concentrations (36).

- **Effective (body) ß2M clearance.**

Effective or body clearance of ß2M was estimated using a two-point kinetic model, based on pre- and corrected post-dialysis concentrations. Post-dialysis values were corrected in two steps: 1. For hemoconcentration, using weight loss as proxy and for extracellular volume contraction; 2 For the compartmentalization effect, incorporating a 60-minute rebound correction as per Tattersall’s method.

- **ß2M mass removal.**

The mass of ß2M removed during each dialysis session was calculated as the product of the time-averaged ß2M concentration (TAC), the effective ß2M clearance (K _ß2M_), and the treatment duration (t_HD_). The result is expressed in milligrams per session. This calculation provides an estimate of the total ß2M solute mass eliminated over the course of a single dialysis treatment.

- **Estimation of equivalent continuous dialytic clearance.**

The equivalent continuous dialytic clearance (eCDC _ß2M_) was calculated using a formula established by the CKD-EPI consortium, which validated ß2M concentration as an accurate marker of glomerular filtration rate (GFR) across all stages of chronic kidney disease (CKD) (34). The equations used is as follows.

GFR = 133 x ß2M^-0.854^

This relationship was derived from a large dataset of CKD patients, including data on serum creatinine, ß2M, and cystatin C, and has been shown to correlate strongly with measured GFR in multiple validation studies.

Notably, Argyropoulos et al. recently confirmed the reliability of this equation through simulated modeling based on a comprehensive meta-analysis dataset of over 10,000 CKD patients (23, 26). Based on the robustness of these findings, we applied this formula as a proxy for estimating equivalent GFR in dialysis patients.
